# Immune profile of squamous metaplasia development in autoimmune regulator-deficient dry eye

**Published:** 2009-03-23

**Authors:** Ying-Ting Chen, Shimin Li, Karina Nikulina, Travis Porco, Marianne Gallup, Nancy McNamara

**Affiliations:** 1 Francis I. Proctor Foundation, University of California, San Francisco, CA; 2 Departments of Epidemiology and Biostatistics, University of California, San Francisco, CA; 3 Departments of Anatomy and Ophthalmology, University of California, San Francisco, CA

## Abstract

**Purpose:**

Squamous metaplasia of the ocular surface epithelium in severe Sjögren syndrome (SS) dry eye has been implicated to be associated with chronic engagement of immune-mediated inflammation. While the detailed immunopathological mechanism underlying keratinization of the ocular muco-epithelium in this setting remains unclear, mice deficient in the autoimmune regulator gene (*Aire*) demonstrate SS-like pathological changes in the exocrine organs and ocular surface including squamous metaplasia. Using this murine model, we sought to determine the specific immune events that predict squamous metaplasia of the cornea in *Aire* deficiency.

**Methods:**

Lissamine green staining, goblet cell density, and corneal small proline-rich protein 1B (SPRR1B) were compared in *Aire*-sufficient and -deficient mice at 4, 8, and 16 weeks of age. Corneal, limbal and conjunctival infiltration of CD4^+^ and CD8^+^ T cells as well as CD11c^+^ and MHC class II (I-A^d+^) dendritic cells (DCs) were examined at the same time points. Ordinary least squares regression was used to model SPRR1B’s relationship with lissamine green staining, goblet cell density, and immune cell infiltration.

**Results:**

Lissamine green staining was present in *Aire*-deficient mice by four weeks of age and increased over time. Compared to *Aire*-sufficient controls, conjunctival goblet cell density (GCD) decreased and corneal SPRR1B increased in *Aire*-deficient mice with significant differences noted at both 8 and 16 weeks. Immune-mediated CD4^+^ T cell infiltration of the conjunctiva and limbus peaked at eight weeks and then decreased. In contrast, corneal T cell infiltration continued to increase over time, reaching a maximum cell number at 16 weeks. CD11c^+^ myeloid-derived DCs were found in the conjunctiva and limbus at all time points. As the mice aged, there was a notable increase in corneal CD11c^+^ cell counts. Interestingly, the dynamic of activated MHC class II^+^ DCs was nearly identical to that of CD4^+^ T cells, peaking first in the limbus at eight weeks with maximum infiltration of the cornea by 16 weeks. Regression analysis showed that squamous metaplasia biomarker, SPRR1B, is strongly related to the lissamine green staining of the ocular surface. Corneal infiltration of activated DCs was most prognostic of corneal SPRR1B expression while the presence of precursor DCs, activated DCs, and CD4^+^ T cells in the limbus were also significant predictors of SPRR1B.

**Conclusions:**

*Aire*-deficient mice represent a useful model to study Sjögren-like autoimmune-mediated ocular surface disease. Results of the current study suggest that squamous cell precursor protein, SPRR1B, provides an important readout to evaluate ocular surface damage and specific events related to immune-mediated inflammation. Results also define an appropriate time frame for interventional studies to develop more effective therapies for keratinizing ocular surface disease.

## Introduction

Dry eye is the most common cause for ophthalmic clinic visits worldwide. In the United States alone, it is estimated that 10 million people are affected by chronic dry eye disease [1]. One of the most debilitating forms of dry eye is the autoimmune-mediated, aqueous-deficient dry eye that occurs in patients with Sjögren’s syndrome (SS). Clinically, SS is an autoimmune disorder characterized by chronic salivary and lacrimal gland inflammation and destruction [2]. The lacrimal hypofunction caused by focal lymphocytic infiltration and destruction of the gland leads to insufficient aqueous production to maintain a stable tear film on the ocular surface. Complaints from patients with SS dry eyes vary from irritation, burning sensation, dryness, unstable/reduced vision, photophobia, grittiness, and sharp, intermittent pain. As SS dry eye progresses, the symptoms can become particularly disabling and refractory to conventional treatment using standard tear supplementation.

A normal, healthy ocular surface consists of a mucus-secreting, non-keratinized, stratified epithelium that stabilizes the tear film and maintains ocular surface homeostasis. In human patients, an important end stage clinical manifestation of chronic dry eye is keratinization of ocular surface epithelium. Keratinization is characterized by the emergence of epidermal-specific keratin K1/K10, cornified envelope proteins, and a concomitant loss of ocular surface mucins in both goblet and non-goblet epithelial cells [3]. This pathological process is known as “squamous metaplasia” of the ocular surface. The severity of dry eye and the degree of squamous metaplasia have been established in human dry eye patients including those with SS [4-6]. Yet, the effector mechanism by which autoimmune-mediated inflammation interacts or leads to squamous metaplasia of the muco-epithelia remains obscure. As a consequence, treatment options to restore vision loss caused by pathological keratinization are limited.

In the setting of dry eye disease, a combination of stress-induced events set off a detrimental cycle of inflammation [7,8]. Based on several lines of in vitro and animal experiments, the Cullen Symposium on Corneal & Ocular Surface Inflammation in 2005 and the Dry Eye Subcommittee of the International Dry Eye Workshop in 2007 proposed a hypothetical mechanism for inflammation in dry eye disease [9,10]. This immune cycle includes an acute stage of three key events: inflammatory mediator release from ocular mucosal epithelial cells, sensitization of  resident immature dendritic cells (DCs) in limboconjunctiva, and eventually centrifugal recruitment of mature DCs-derived MHC class II^+^ antigen-presenting cells (APCs) from limboconjunctiva to the cornea. With persistent stress, chronic immune inflammation ensues with the procurement and presentation of antigen by APCs that travel to regional lymph nodes where they prime naïve T-cells. Primed and targeted CD4^+^ T cells home to the ocular surface, causing damage to the lacrimal functional unit, an integrated system that includes the cornea, conjunctiva, and lacrimal gland. This damage causes a remarkable decrease in tear-binding mucins (MUC5AC, MUC19) [11,12], reduced goblet cell density (GCD) [13], and an increase in cornified envelop precursors such as small proline rich proteins (SPRRs) [14,15]. While chronic inflammation has been implicated in the onset of squamous metaplasia, more specific details regarding the dynamics of the immune response and the importance of other immune cell populations as potential predictors of squamous metaplasia are not available.

An animal model that mimics the pathogenesis of SS dry eye would be an ideal tool to study the immunopathology of autoimmune-mediated aqueous deficiency. Recently, we and others have characterized the ocular surface pathology of a murine model that mimics the multifaceted pathological changes of SS [15-17]. Mice that are deficient in the autoimmune regulator gene (*Aire*) have a failure in central tolerance that leads to autoreactive thymocytes, causing multi-organ autoimmune disease. In this model, both the inflammatory and squamous metaplastic features of SS dry eye are demonstrated. Previously, we showed a definitive link between CD4^+^ T cell activation and pathological keratinization of the ocular surface in *Aire*-deficient mice using a biomarker of squamous metaplasia, small proline-rich protein 1B (SPRR1B). Increased SPRR1B was observed in impression cytology samples of human patients with SS, in *Aire*-deficient mice, and in *Aire*-sufficient severe combined immunodeficiency (SCID) mice following adoptive transfer of *Aire*-deficient CD4^+^ T lymphocytes [15].

To further explore clinical applications for the *Aire*-deficient murine model of dry eye and in preparation for interventional studies to block the onset of pathological keratinization, we profiled the onset and dynamics of immune-mediated inflammation and squamous metaplasia. We show the dynamics of lissamine green staining, SPRR1B expression, goblet cell loss, and immune cell infiltration over time in the setting of *Aire* deficiency. We demonstrate a compelling link between squamous metaplasia and immune cell infiltration by modeling the relationship of SPRR1B with markers of ocular surface damage and inflammation. These findings demonstrate the utility of using SPRR1B to assess the state of immune cell infiltration, the usefulness of *Aire*-deficient mice for studying the biological course of autoimmune-mediated dry eye, and the appropriate time frame for interventional studies to develop more effective therapies in dry eye disease.

## Methods

### Materials

All materials used were purchased from Sigma (St. Louis, MO) unless otherwise indicated. The 16% formaldehyde solution was obtained from Thermo Fisher Scientific Inc. (Waltham, MA), the microscope slides were Superfrost/Plus from Fisher Scientific (Pittsburgh, PA), and the optimal cutting temperature (OCT) compound was obtained from Sakura (Tokyo, Japan). The kit for periodic acid-Schiff (PAS) was purchased from American Master Tech Scientific, INC. (Lodi, CA), the 3,3'-Diaminobenzidine (DAB) substrate kit for peroxidase was from Vector Laboratories (Burlingame, CA), hematoxylin was from Richard-Allan Scientific (Kalamazoo, MI), and DAPI (4',6'-diamino-2-phenylindole) was from Molecular probes (Eugene, OR). Lissamine green dye was obtained from Leiter’s Pharmacy and Compounding Center (San Jose, CA).

### Mice

Mice deficient in *Aire* (BALB/c *Aire*^−/−^; a gift of Dr. Mark Andersen, University of California, San Francisco [UCSF], CA) and age-matched heterozygous controls (+/−) were studied from 4−16 weeks of age (n=minimum of four per age group). Mice were housed in a pathogen-free barrier facility at the University of California, San Francisco. Offspring mice were genotyped for the *Aire* mutation by polymerase chain reaction (PCR) of their genomic DNA. The dry eye phenotype was assessed through topical application of lissamine green dye (1%) to detect keratinized and devitalized epithelial cells on the ocular surface [18] without the need for cobalt light excitation. Surface damage of the eye was imaged using a Nikon Coolpix5400 digital camera (Nikon Instruments Inc., Melville, NY) fitted to Olympus Zoom Stereo Microscope (Olympus America Inc., Center Valley, PA) and was graded using a modification of the van Bijsterveld grading system [19]. Briefly, a masked, well trained observer scored the slides on a scale as follows: 0, no staining; 1, less than 25% of cornea stained with scattered punctate staining; 2, 25%–50% of cornea  stained with diffuse punctate staining; 3, 50%–75% of cornea  stained with punctate staining and apparent epithelial defects; and 4, more than 75% of cornea stained with abundant punctate staining and large epithelial defects. The mice were euthanized by carbon dioxide inhalation and thoracotomy followed by cardiac perfusion with 0.1 M PBS. All animal procedures were approved by the Institutional Animal Care and Use Committee at the University of California, San Francisco and firmly adhered to the ARVO Statement for the Use of Animals in Ophthalmic and Vision Research.

### Histology, immunofluorescence, and immunostaining

Lacrimal glands, eyes, and adnexa were dissected and embedded in OCT (for immunostaining) or fixed in 10% phosphate-buffered formalin and embedded in paraffin (for histology). Immune cell types were visualized by immunostaining as previously described [20]. The following antibodies were used at 1:50 dilution for 1 h at room temperature: anti-CD4, anti-CD8, anti-CD11c, and anti-I-A^d^ (all from BD PharMingen, San Diego, CA). Horseradish peroxidase (HRP)-conjugated secondary antibody was applied for 30 min (Jackson ImmunoResearch Laboratories, West Grove, PA). Pre-diluted streptavidin-HRP was used for I-A^d^ staining (BD PharMingen). Samples were incubated with peroxidase substrate and counterstained with hematoxylin. For immunofluorescence, sections were exposed to primary anti-SPRR1B antibody at a 1:800 dilution (gift from Dr. Reen Wu, University of California, Davis) followed by FluoroLink Cy3-conjugated secondary antibody (Amersham Bioscience, Piscataway, NJ). Nuclei were counterstained with DAPI. Negative controls included the omission of the primary antibody or isotype control. Three sections from each animal were examined. Goblet cells in the tarsal conjunctiva and fornix were counted by a masked, well trained observer in three paraffin-embedded sections from one eye of at least six animals stained with PAS.

### Quantification of SPRR1B and immune cells

All images were acquired using NIS Elements BR2.30 software (Nikon Instruments Inc.) with a Retiga 2000R digital camera (QImaging, Surrey, BC, Canada) fitted on a Nikon Microphot SA microscope (Nikon Instruments, INC.) or using Spot Advanced 4.0.9 software (Diagnostic Instruments, Sterling Heights, MI) with an RT Slider Spot camera (Diagnostic Instruments) fitted on a Nikon Eclipse E800 microscope (Nikon Instruments Inc.). Fluorescence measurements of SPRR1B staining obtained using the red (Cy3) channel were converted to an 8-bit gray scale image (fluorescence intensity range, 0-255) and analyzed using Image J 1.40 g software [21]. Digital images of the central cornea were acquired at 200X. Regions of interest (ROI), which were defined by tissue boundaries using the DAPI staining pattern, were outlined with the freehand tool of Image J. Fluorescence intensities of the pixels within the ROI were recorded with Image J and normalized to the negative control. Results were expressed as averages of the mean fluorescence intensity per ROI. A minimum of six eyes were analyzed at each time point for *Aire*^−/−^ and *Aire*^+/−^ mice. Immune cell populations stained with DAB were analyzed in the central cornea, tarsal conjunctiva, and limbus. A masked, well trained observer quantified each cell type at 200X magnification in three sections of a single eye from at least four different animals. Positively stained cells were counted in the limbus, conjunctiva, and central cornea using NIS Elements BR2.30 image-analysis software, which is routinely used for quantification of the DAB staining [22,23]. Positive areas were distinguished from the background by applying threshold values for ROI. Results are expressed as the total area effected by DAB staining per ROI.

### Statistics

Results are presented as mean±standard deviation of the mean (SD). We used a student *t*-test to compare differences in lissamine green staining, goblet cell density, corneal SPRR1B, and CD4^+^, CD8^+^, CD11c^+^ and I-A^d+^ cell counts between knockout and heterozygous mice at three different ages. We adjusted for multiple comparisons using the standard Holm procedure. We modeled the relationship between corneal SPRR1B expression and markers of ocular surface damage and inflammation using ordinary least squares regression (transforming both independent and dependent variables to achieve normality and homoscedasticity and to reduce the influence of specific observations). Homoscedasticity was assessed using standard residual plots. Normality of residuals was assessed by the Anderson-Darling procedure. The leave-one-out influence statistic was used to assess the presence of highly influential observations. We only included quadratic terms in the regression models when these terms were statistically significant (assessed by the T statistic for the coefficient of the quadratic term). We tested the hypothesis that corneal expression of SPRR1B did not depend on each marker of ocular surface damage and inflammation (i.e., the linear term and quadratic term were both zero) using the F-test. All analyses were done using the statistical software, Stata 9.0 (College Station, TX) or R 2.8.1 for MacIntosh (Cupertino, CA).

## Results

### Lacrimal gland and ocular surface damage in *Aire*-deficient mice

**Figure 1:**
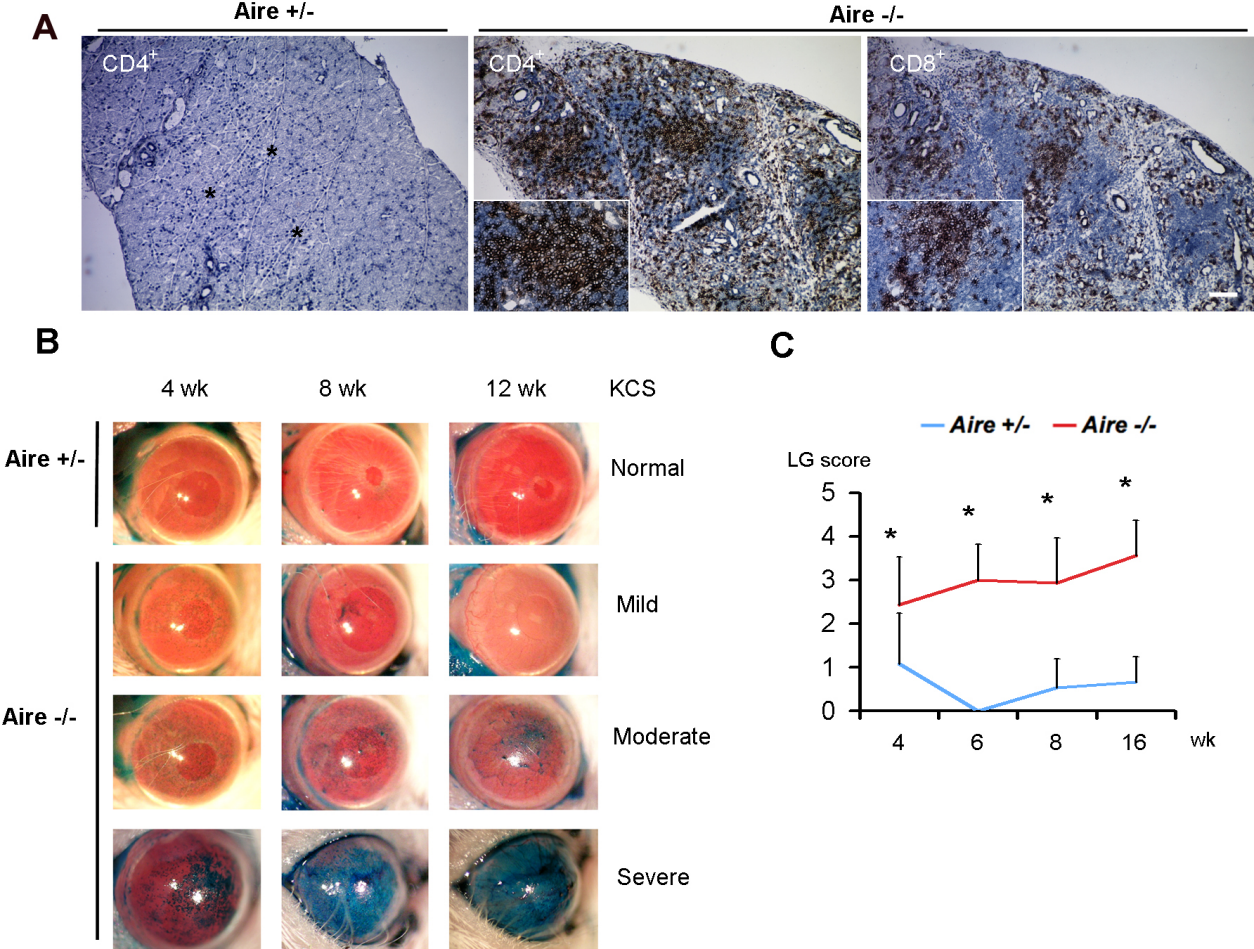
Infiltration and destruction of the lacrimal gland and ocular epithelium in *Aire*-deficient mice. **A**: Immunohistochemical analysis of CD4^+^ and CD8^+^ T cells was negative in *Aire*^+/−^ while *Aire*^−/−^ mice showed intensive multifocal aggregates of both cell types. Of note, characteristic interlobular connective tissue that exists in the normal lacrimal gland (asterisks) disappears in the *Aire*^−/−^ lacrimal gland following lymphocytic infiltration. Bar, 100 μm. **B**: Time course of lissamine green staining in the corneas of *Aire*^+/−^ and *Aire*^−/−^ mice demonstrates a compromised epithelial integrity caused by Aire deficiency. Mild to severe keratoconjunctivitis sicca (KCS) shown as punctate to confluent green staining were observed in *Aire*^−/−^ mice whereas corneas of *Aire*^+/−^ largely remained unstained. **C**: Progression of lissamine scores is shown on a chart. Data are shown as mean±SD. The asterisk in this panel indicates that p<0.05, *Aire*^+/−^ versus *Aire*^−/−^ at each time point.

To validate the *Aire*-deficient mouse as an appropriate model for Sjögren syndrome-like dry eye in humans, we looked for the presence of lacrimal gland inflammation and destruction. In *Aire*-sufficient mice (*Aire*^+/−^), histology of the lacrimal gland showed characteristics of normal structures such as well defined acinar cell borders, apparent extracellular spaces, and secretory ducts (Figure 1A, *Aire*^+/−^). Immunostaining for CD4^+^ and CD8^+^ T lymphocytes was negative (CD4^+^ cells, Figure 1A, *Aire*^+/−^; CD8^+^cells, data not shown). In contrast, acinar cell atrophy and small duct dilation indicated significant damage to the gland in *Aire*-deficient mice (*Aire*^−/−^) with extensive cellular infiltration as early as four weeks of age (Figure 1A, *Aire*^−/−^). Immunostaining revealed mononuclear infiltrating cells to be predominantly CD4^+^ T cells and to a lesser extent CD8^+^ cells. To evaluate the state of ocular damage, lissamine green staining was monitored in age-matched 4-, 6-, 8-, 12-, and 16-week-old *Aire*^+/−^ and *Aire*^−/−^ mice. Figure 1B shows representative progression of ocular surface damage as a function of time in the eye of one heterozygous mouse (top row) and three knockout mice (rows 2–4) over a period of 12 weeks. Four degrees of dry eye were observed: Normal, free of lissamine green staining; Mild, sparse punctate staining without progression; Moderate, numerous punctate staining with progression to coalesce central staining; Severe, coalesced central staining with progression to epithelial opacification. Out of 17 *Aire*-deficient and 16 *Aire*-sufficient mice, none of the knockouts were free of lissamine green staining whereas 47% of heterozygous mice (*Aire*-sufficient) showed no staining. Mild, moderate, and severe dry eye were respectively found in 19%, 31%, and 50% of *Aire*^−/−^ mice compared to 41%, 12%, and 0% for *Aire*^+/−^. As shown in Figure 1C, the difference in staining was significant as early as four weeks and increased with time. Data are summarized in Table 1.

**Table 1:** Lissamine green staining, SPRR1B expression, and goblet cell density.

**Ocular surface marker**	**Genotype**	**Central cornea**			
		**4 wk**	**6 wk**	**8 wk**	**16 wk**
Lissamine	*Aire*^+/−^	1.08±1.16	0±0	0.54±0.66	0.67±0.58
	*Aire*^−/−^	2.44±1.09	3±0.82	2.93±1.03	3.57±0.79
		**Central cornea**			
		**4 wk**	**8 wk**	**16 wk**	
SPRR1B	*Aire*^+/−^	49.41±14.19	52.97±8.92	52.19±22.26	
	*Aire*^−/−^	67.6±17.56	98.77±23.41	82.52±25.17	
		**Conjunctiva**			
		**4 wk**	**8 wk**	**16 wk**	
Goblet Cell Density	*Aire*^+/−^	140.29±18.17	128.91±20.39	131.57±41.13	
	*Aire*^−/−^	108.0±38.72	82.09±49.13	83.77±59.25	

Data are shown as mean±SD in arbitrary units. Wk = week

### Pathological keratinization of the ocular mucosal epithelium in *Aire*-deficient mice

**Figure 2:**
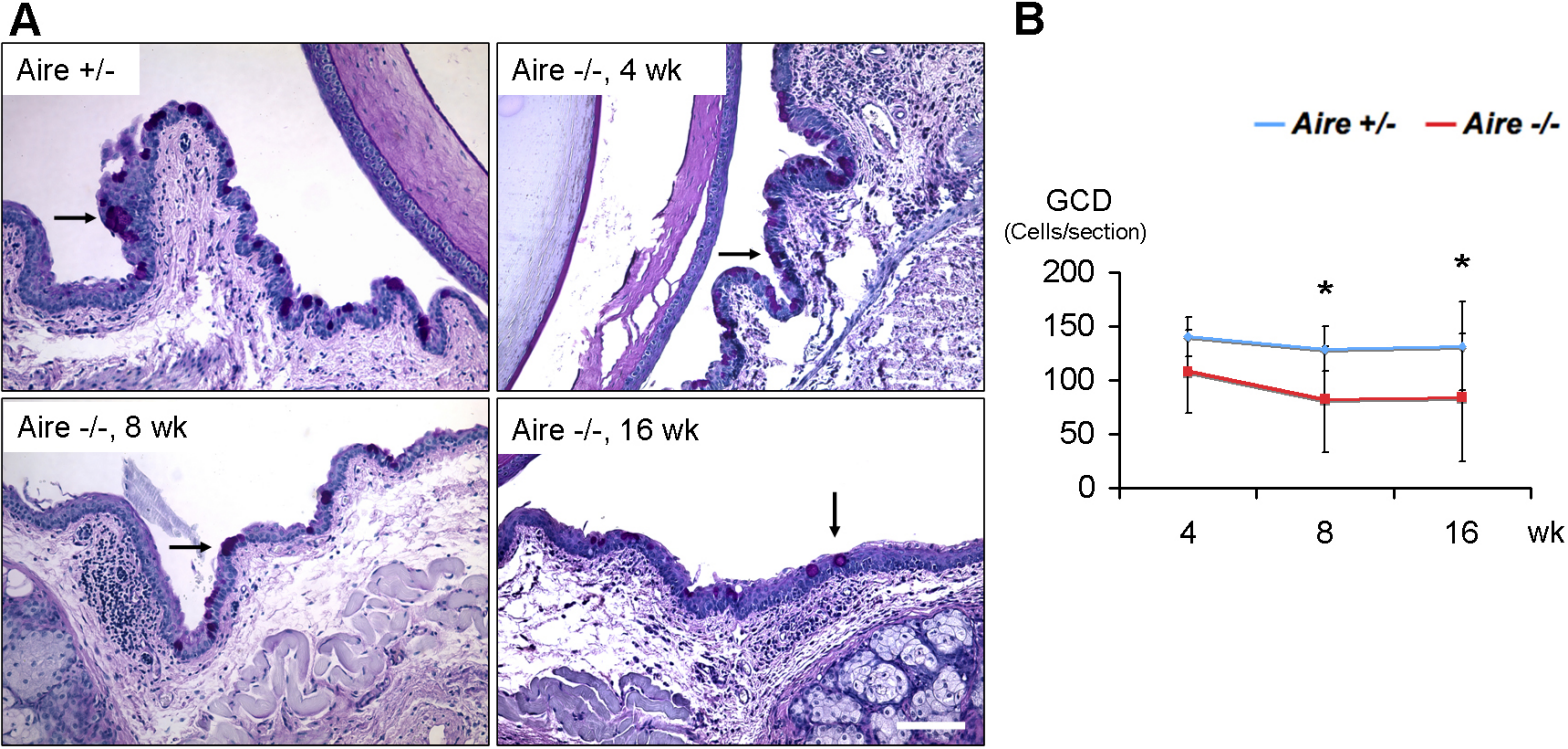
Time course of goblet cell density in *Aire*^+/−^ versus *Aire*^−/−^ mice. **A**: Periodic acid–Schiff staining revealed abundant goblet cells (arrow) in *Aire*^+/−^ at all time points (representative example shown) and in *Aire*^−/−^ at four weeks of age. A gradual decrease in goblet cell density was observed in the conjunctiva of *Aire*^−/−^ mice over time. Bar, 100 μm. **B**: Time course of goblet cell density is shown as mean±SD. An asterisk indicates that p<0.05, *Aire*^+/−^ versus *Aire*^−/−^ at each time point.

As shown previously, goblet cell loss and increased expression of cornified envelope precursor proteins (e.g., SPRR1B, SPRR2) denotes pathological keratinization of the mucus-secreting ocular surface epithelium [14,15]. To further assess damage in *Aire*-deficient mice, we examined the abundance of conjunctival goblet cells and the level of corneal SPRR1B. Goblet cells were reduced as early as four weeks of age in *Aire*-deficient mice with a notable decrease between four and eight weeks. Goblet cells were significantly reduced at 8 and 16 weeks compared to *Aire*^+/−^ mice (Figure 2A,B).

**Figure 3:**
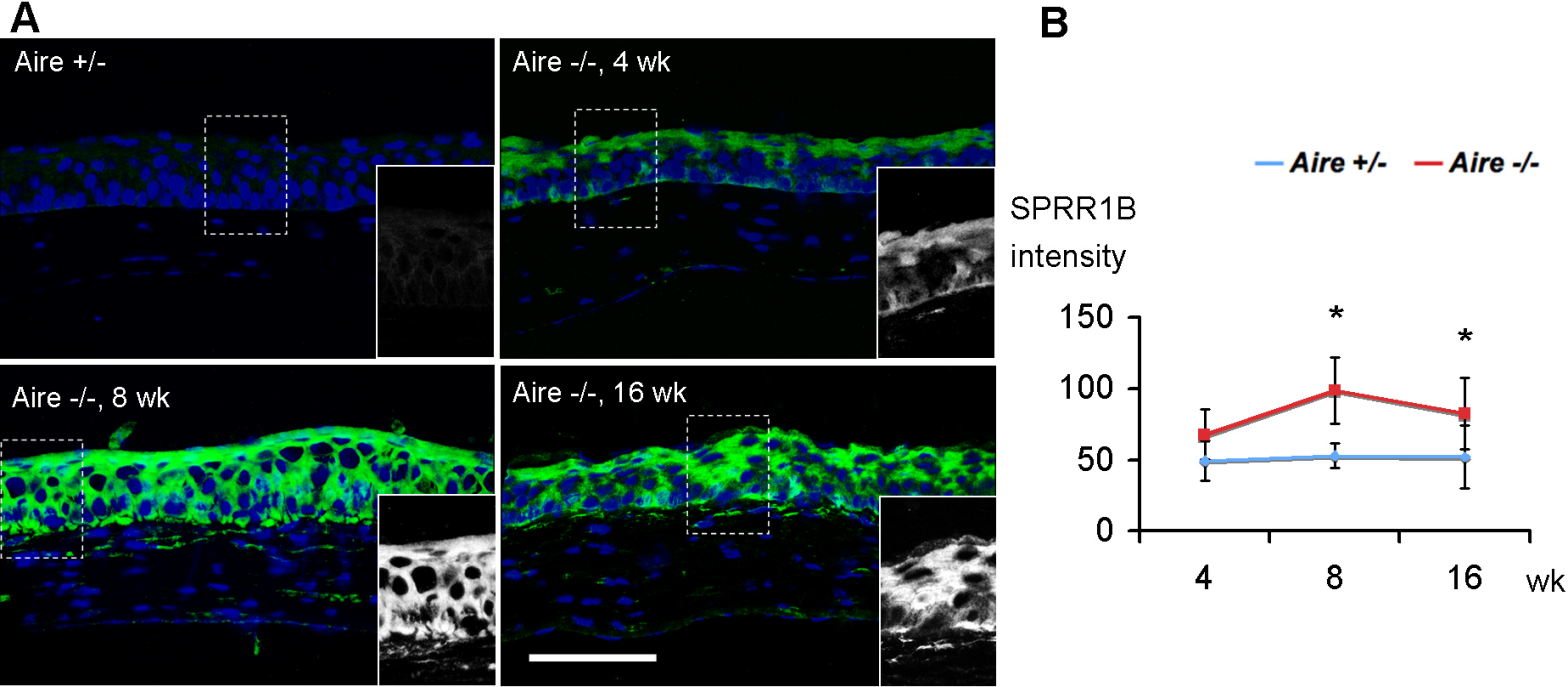
Expression of squamous cell biomarker, SPRR1B. **A**: Immunofluorescence showed increased SPRR1B staining (green) in *Aire*^−/−^ at all time points with no staining in age-matched *Aire*^+/−^ controls. Bar, 100 μm. **B**: Quantification of SPRR1B immuno-intensity over time. Data are shown as mean±SD. An asterisk in indicates that p<0.05, *Aire*^+/−^ versus *Aire*^−/−^ at each time point.

In conjunction with goblet cell drop out, corneal SPRR1B expression was increased in *Aire*-deficient mice. Superficial expression of SPRR1B was present at four weeks and progressed to a more intense full thickness staining at eight weeks in the presence of epithelial hyperplasia. Increased levels of corneal SPRR1B persisted to 16 weeks while the corneal epithelium progressed to metaplasia. By comparison, *Aire*-sufficient mice showed minimal to no expression of SPRR1B at all time points (Figure 3A,B). Together, goblet cell drop out and increased corneal SPRR1B indicate the presence of ocular surface keratinization by eight weeks in *Aire*-deficient mice, and these changes persist with age. Data are summarized in Table 1.

### Immune cell infiltration of the conjunctiva, limbus, and cornea in *Aire*-deficient mice

**Figure 4:**
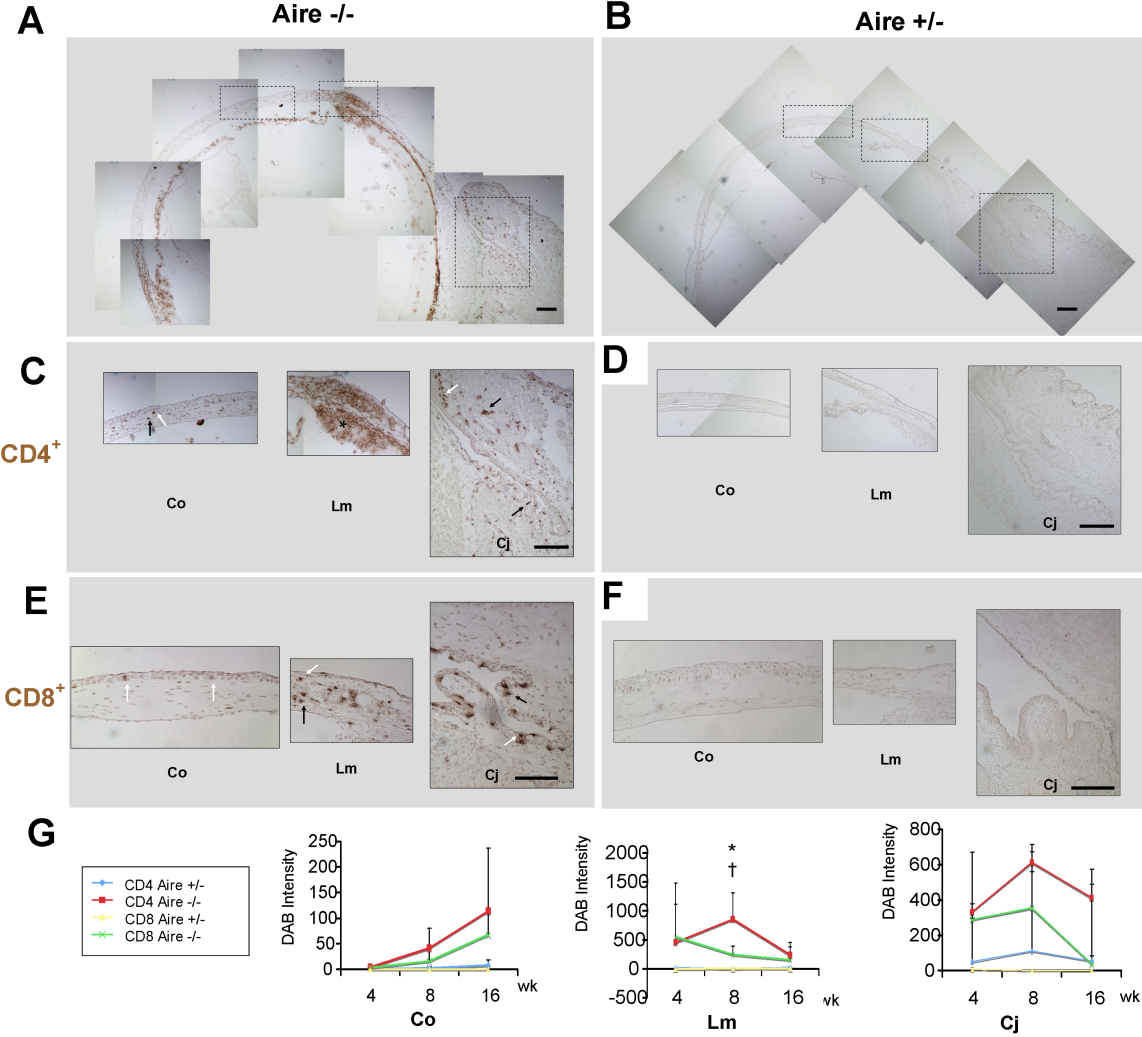
T cell distribution in the ocular surface of *Aire*-deficient mice. Assemblage of low magnification images showing anatomic locations analyzed in *Aire*^−/−^ (**A**) and *Aire*^+/−^ (**B**) mice. Cj=conjunctiva; Lm=limbus; Co=cornea (paracentral and central). Immunohistochemistry of CD4^+^ (**C**,**D**) and CD8^+^ T (**E**,**F**) cells demonstrates both epithelial (white arrow) and stromal (black arrow) infiltration of the ocular surface in *Aire*^−/−^. *Aire*^+/−^ controls exhibited no cell-mediated inflammation at the locations analyzed. Bar, 100 μm. **G**: Time course of CD4^+^ and CD8^+^ cell density in *Aire*^−/−^ and *Aire*^+/−^. Data are shown as mean±SD. An asterisk indicates that  p<0.05, CD4^+^ *Aire*^+/−^ versus CD4^+^ *Aire*^−/−^. The symbol, †, indicates that p<0.05, CD8^+^ *Aire*^+/−^ versus CD8^+^ *Aire*^−/−^.

In previous work, we defined a specific role for autoreactive T cells as inducers of squamous metaplasia [15]. The current work provides an important connection between inflammation and keratinizing ocular surface disease while demonstrating the usefulness of SPRR1B as a surrogate biomarker for squamous metaplasia. To better define the immune cell populations that exist in the *Aire*-deficient mouse and their relationship to corneal SPRR1B, we examined the localization and infiltration of four different immune cell populations, CD4^+^ helper T lymphocyte, CD8^+^ cytotoxic T lymphocyte, CD11c^+^ myeloid-derived dendritic cells, and activated dendritic cells expressing the MHC class II alloantigen (I-A^d^) [24-27]. A minimum of four heterozygous and four knockout mice were included to assess the distribution of each cell type at three different time points (4, 8, and 16 weeks). Figure 4A demonstrates representative CD4^+^ T cell distribution throughout the ocular surface of *Aire*-deficient mice while confirming the absence of T cells in heterozygous controls (Figure 4B). The density of each cell type was quantified in three different locations, the tarsal conjunctiva (Cj), the limbus (Lm), and the cornea (Co). CD4^+^ (Figure 4C) and CD8^+^ (Figure 4E) T cells were found throughout the ocular surface of *Aire*-deficient mice with CD4^+^ cells being the most abundant and most intensely localized in the limbus. Both T cell populations were concentrated in 1) the epithelium and superficial substantia propria of the conjunctiva, 2) the epithelium and stroma of the limbus, and 3) the subepithelial stroma of the central cornea (Figure 4C,E). By comparison, there is no noticeable infiltration of either cell type in *Aire*-sufficient mice (Figure 4D,F). Over time, CD4^+^ infiltration of the conjunctiva and limbus increased from four to eight weeks and slowly declined before reaching 16 weeks. As CD4^+^ cells began to decrease in the conjunctiva and limbus at eight weeks, corneal infiltration steadily increased and finally peaked by the 16th week (Figure 4G, red line). In general, the infiltration of CD8^+^ cells followed the same pattern, although the degree of inflammation was considerably less (Figure 4G, green line). Taken together, data here indicate predominantly CD4^+^ T cell infiltration of the ocular surface in *Aire*-deficient ocular surface.

**Figure 5:**
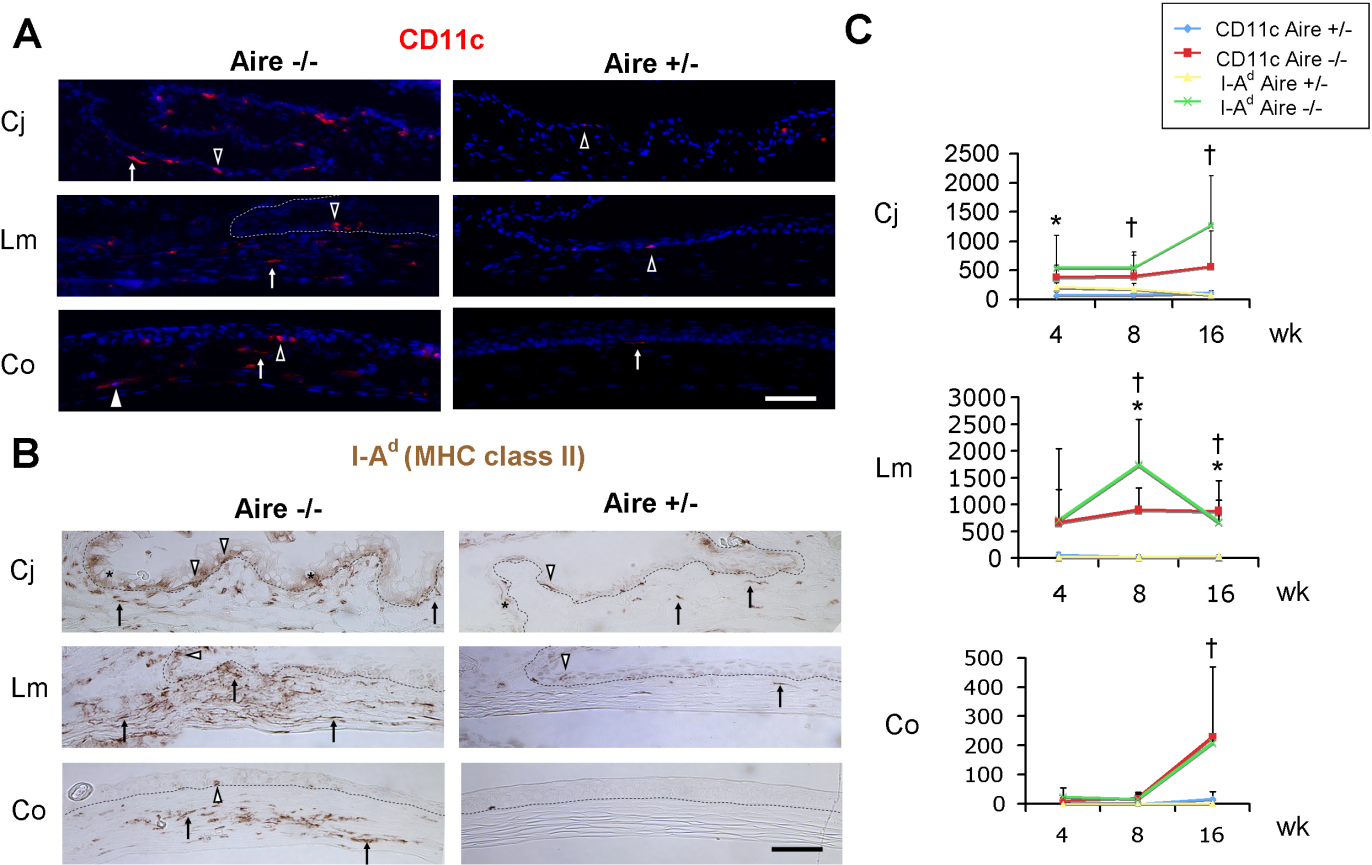
Dendritic cell distribution across the ocular surface of *Aire*-deficient mice. **A**: Immunofluorescence study of CD11c^+^ cells (red) demonstrates dendritic antigen presenting cells (APCs) in the basal epithelium (open arrowhead) and sub-epithelial stroma (arrow) of the conjunctiva (Cj), limbus (Lm), and central cornea (Co). Although CD11c^+^ cells are found in both *Aire*^+/−^ and *Aire*^−/−^ mice, significantly more are seen in *Aire*-deficient mice. CD11c^+^ cells are present throughout the whole layer of the cornea including the posterior stroma (solid arrow head) in *Aire*^−/−^ while they are largely absent in the posterior cornea in *Aire*^+/−^. The dotted line represents the epithelial basement membrane in the limbus, and the blue nuclear counterstaining (DAPI) is used for orientation. Bar, 100 μm. **B**: Immunohistochemical study of MHC class II surface antigen (I-A^d^) reveals an intense infiltration of activated dendritic APCs in the epithelium (open arrow head) and stroma (arrow) of the conjunctiva (Cj), limbus (Lm), and central cornea (Co) of *Aire*^−/−^ mice. By comparison, I-A^d+^ cells are rarely apparent in the conjunctiva and limbus and are completely absent in the central cornea of *Aire*^+/−^ mice. The asterisk denotes goblet cells. Bar, 100 μm. **C**: Quantification of CD11c+ and I-A^d+^ cells is shown over time. Data are shown as mean±SD. An asterisk indicates that p<0.05, CD11c^+^ *Aire*^+/−^ versus CD11c^+^ *Aire*^−/−^. The symbol, †, indicates that p<0.05, I-A^d+^ *Aire*^+/−^ versus I-A^d+^ *Aire*^−/−^.

Next, we examined the presence and localization of APCs that begin the immunological loop that ultimately leads to the presence of primed, effector T cell infiltration into the ocular surface. Myeloid CD11c^+^ DCs are known as resident APCs in the normal ocular surface [26,27]. These sentinel cells were found throughout the ocular epithelium in all three anatomic locations. However, the distribution and density differed in the presence and absence of Aire. In *Aire*-deficient mice, the infiltration of CD11c^+^ DCs was intense and throughout the full thickness of the ocular epithelia. By comparison, in *Aire*-sufficient mice, the CD11c^+^ DCs were much less in number and the majority of cells were localized in the epithelium and substantia propria of the conjunctiva with a minor number in the epithelium and subepithelial stroma of the limbus and cornea (Figure 5A, *Aire*^+/−^). In *Aire*-deficient mice, CD11c^+^ cells were initially concentrated in the limbus with the overall cell count peaking at eight weeks. Over the next eight weeks, limbal CD11c^+^ cells decreased while the number of corneal CD11c^+^ cells increased. These results suggest centripetal movement of DCs into the cornea from their limbal residency in the presence of chronic inflammation (Figure 5A,C, *Aire*^−/−^). An antibody directed against the MHC class II molecule, I-A^d^, was used to examine the distribution of activated, antigen-presenting DCs in the Balb/c mouse [26]. In *Aire*-sufficient controls, a small number of I-A^d+^ cells were identified in the epithelium and stroma of the conjunctiva and limbus. There were no activated DCs in the cornea (Figure 5B, *Aire*^+/−^). By comparison, I-A^d+^ cells infiltrated all areas of the inflamed ocular surface including the epithelium and stroma of the conjunctiva, limbus, and cornea (Figure 5B, *Aire*^−/−^). Quantification of DCs revealed significant increases in both the overall and active populations (CD11c^+^ and I-A^d+^, respectively) as the mice aged. Activated cells peaked in the limbus at eight weeks and then declined. As limbal cell counts decreased, there was a robust increase in corneal DCs, suggesting centripetal movement of these cells from the limbus into the cornea as inflammation persisted (Figure 5C). This pattern was identical to that observed with the effector T cell populations (Figure 4G). Data are summarized in Table 2. Together, these data suggest increased activity of both afferent and efferent arms of the immune cycle. As inflammation progresses, the processes of corneal antigen presentation by I-A^d+^ cells and resulting corneal infiltration of effector T cell complete the loop that drives ocular surface damage in the setting of chronic inflammation.

**Table 2:** Profile of anterior segment immune cells in *Aire*-sufficient versus *Aire*-deficient mice.

**Immune cell**	**Genotype**	**Central Cornea**			**Limbus**			**Conjunctiva**		
		**4 wk**	**8 wk**	**16 wk**	**4 wk**	**8 wk**	**16 wk**	**4 wk**	**8 wk**	**16 wk**
CD4^+^	*Aire*^+/−^	1.03±2.06	2.26±2.62	8.34±9.84	13.96±21.98	2.62±3.80	18.85±22.99	50.46±47.73	111.58±60.24	55.24±76.48
	*Aire*^−/−^	4.06±3.73	41.53±38.61	113.97±122.86	455.42±657.03	854.13±455.42	235.55±218.41	332.03±247.28	611.79±602.86	411.92±518.64
CD8^+^	*Aire*^+/−^	0±0	0±0	0±0	0±0	3.123±6.25	6.94±13.89	6.39±12.77	0.72±1.43	0±0
	*Aire*^−/−^	3.43±6.86	16.33±17.11	67.45±104.51	551.35±928.04	247.58±144.59	151.84±224.85	289.6±380.37	355.02±204.18	38.13±46.22
CD11c^+^	*Aire*^+/−^	0.18±0.36	0.06±0.13	17.44±24.26	58.79±57.03	24.12±48.00	27.59±18.75	79.83±38.66	80.5±71.92	117.86±116.45
	*Aire*^−/−^	11.57±8.83	21.26±18.95	230.96±239.18	676.25±607.16	906.94±401.30	881.14±567.89	382.78±205.78	403.39±353.03	565.99±608.88
I-A[d]^+^	*Aire*^+/−^	0±0	0.322±0.645	0.45±0.9	26.95±36.69	22.47±20.22	44.57±34.75	213.91±70.22	181.41±92.18	85.84±69.44
	*Aire*^−/−^	24.90±29.25	17.85±15.30	210.99±19.59	714.18±1329.62	1746.79±845.55	687.06±401.39	543.61±557.55	552.40±264.02	1280.06±845.53

Data are shown as mean±SD in arbitrary units. Wk = week

### SPRR1B expression is related to the activation of afferent and efferent arms of the immune cycle

To demonstrate the relationship between chronic inflammation and keratinizing ocular surface disease, we examined the utility of using SPRR1B as a clinical biomarker to follow the progression of ocular surface damage and immune cell infiltration in *Aire*-deficient mice. To do this, we modeled the relationship between corneal SPRR1B and markers of ocular surface damage (i.e, lissamine green staining and goblet cell density) and immune cell infiltration (i.e., CD4^+^, CD8^+^, CD11c^+^, and I-A^d+^). These studies allowed us to examine the hypothesis that SPRR1B expression was dependent on the extent of ocular surface damage and the level of immune cell infiltration.

**Figure 6:**
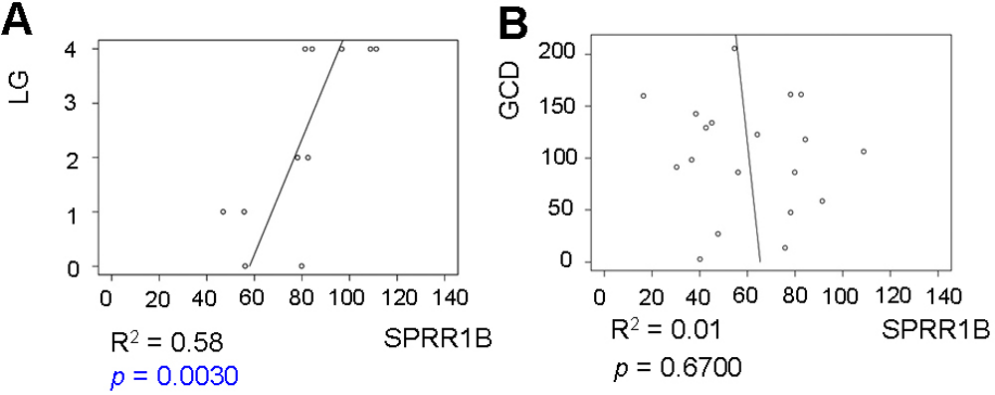
Corneal SPRR1B expression is related to lissamine green staining but not goblet cell density. **A**: Fifty-eight percent of the variability in corneal SPRR1B (expressed as mean intensity score) is explained by the amount of lissamine green staining (LG staining; R^2^=0.58, p=0.0030, n=11) versus only 1% by goblet cell density (GCD) in the right panel (**B**; R^2^=0.01, p=0.67, n=19). Open circles represent raw data, and the x-axis plots SPRR1B immuno-intensity in arbitrary units while the y-axis plots the lissamine green score (**A**) or GCD (**B**; cells/section). The straight line is the best-fit linear regression (ordinary least squares)

As anticipated, there was a strong and statistically significant relationship between corneal SPRR1B and lissamine green staining of the cornea (Figure 6A, p<0.05). In contrast, corneal SPRR1B was not related to goblet cell density (Figure 6B, p=0.67). This is interesting as both the appearance of cornified envelope precursors such as SPRR1B and the disappearance of goblet cells occur in the process of squamous metaplasia. The strength of the relationship between SPRR1B and lissamine green suggests that SPRR1B and other precursor proteins mark the early stages of squamous metaplasia whereas loss of goblet cells tends to be a late stage consequence of chronic inflammation.

**Figure 7:**
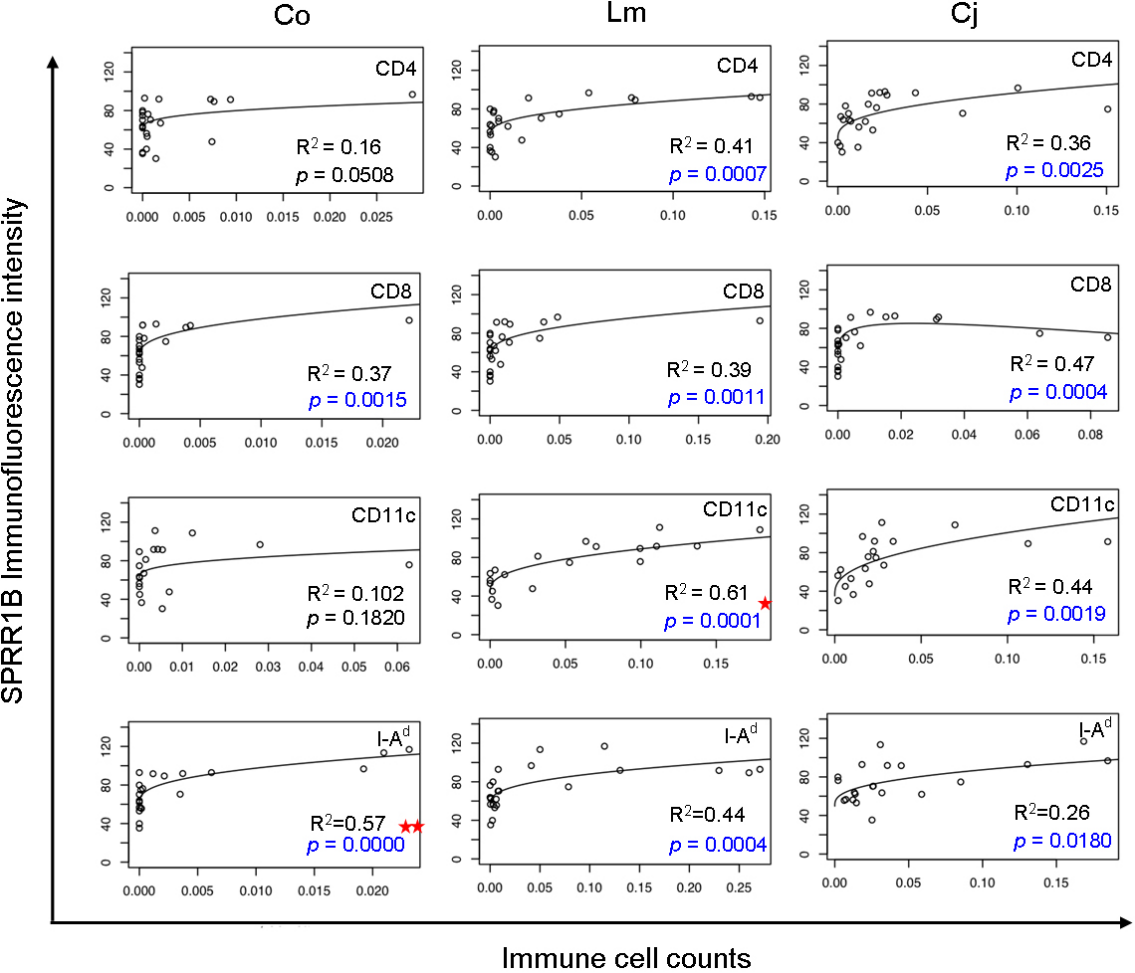
Immune cell infiltration of the ocular surface predicts corneal SPRR1B expression. For each of the 12 subfigures, we plotted SPRR1B as fluorescent intensity on the y-axis in arbitrary units while immune cell counts (CD4, CD8, CD11c, and I-A^d^) are plotted on the x-axis in three different locations (Co, cornea; Lm, limbus; Cj, conjunctiva). Each graph shows raw observations (open circles) as well as a regression line. Each regression line was fit using ordinary least squares to ensure the validity of statistical tests with respect to normality. In order to minimize highly influential points, we transformed both X and Y using the cube root and logarithm, respectively. Curvilinear graphs appear when the regression model is transformed back to the original scale. Centered quadratic terms were included only when statistically significant at the 0.05 level, which only occurred for CD8 cells in the conjunctiva, row 2, column 3. Significant p values (shown in blue) were computed to test the null hypothesis that SPRR1B did not depend on each of the 12 immune cell counts. Note that these p values were adjusted for multiple comparisons using the Holm method. Squared multiple correlation coefficients indicate the fraction of the variance explained by the regression model. The most compelling predictors of corneal SPRR1B were corneal I-A^d+^ cells (two stars) and limbal CD11c^+^ cells (one star). CD4^+^ and I-A^d+^ immune cells in the limbus as well as generalized presence of immune cells in the conjunctiva also showed strong and statistically significant relationships to SPRR1B expression.

The relationship between SPRR1B and immune cell infiltration of the ocular surface is summarized in Figure 7. Levels of SPRR1B in the cornea were highly dependent on the presence and localization of several immune cell populations. The most compelling predictors of SPRR1B expression were the presence of resident DCs in the limbus and activated DCs in the cornea (p<0.0001). SPRR1B expression was also largely dependent on the presence of activated DC and CD4^+^ T cell populations in the limbus (p≤0.0007). Together, data presented here imply a close relationship between the APC, effector T cells, and SPRR1B expression.

## Discussion

Autoimmune diseases such as Sjögren syndrome are mediated by sustained adaptive immune responses specific for self-antigens through an unknown pathogenic mechanism. Although breakdown of self-tolerance is considered to be the key event in the disease process, the mechanisms that allow the production of autoreactive lymphocytes and/or auto antibodies are largely enigmatic [28]. Thus, pertinent animal models are needed to study the complicated pathophysiology.

The autoimmune regulator gene (*AIRE*; OMIM 240300) encodes the Aire protein, which bears strong resemblance to a transcription factor localized in a subset of medullary thymic epithelial cells that is associated with negative selection of developing thymocytes (reviewed in [29]). Loss of *Aire* expression results in an inability to remove autoreactive thymocytes from the immune repertoire, and this leads to the development of a population of mature, autoreactive T cells capable of mediating multi-organ autoimmune disease [17,20]. *Aire*-deficient mice show autoreactive T cell migration into the exocrine glands that mimics the pathology of SS [16,17]. Yet, the dynamics of ocular surface damage as a result of lacrimal gland destruction have never been studied in this animal model.

Data presented in the current study validate the *Aire*-deficient Balb/c mouse as an appropriate model to study Sjögren-like dry eye disease. Characteristics of lacrimal gland destruction and multifocal lymphocyte aggregation resembled the clinical exocrine gland biopsy results of human patients with Sjögren syndrome (Figure 1A). Infiltrating lymphocytes in *Aire*-deficient lacrimal glands were mainly T cells (both CD4^+^ and CD8^+^ subsets, Figure 1A). This phenotype mimics the clinical histopathology of human exocrine glands of SS patients, which contain predominantly CD4^+^CD45RO^+^ve cells [30,31] and cytotoxic CD8^+^CD103^+^ve cells [31], with only up to 20% of infiltrating lymphocytes identified as B cells [32]. In conjunction with lacrimal gland destruction, there is significant compromise to the ocular surface mucosal epithelium in *Aire*-deficient mice. Punctate to diffuse lissamine green staining was routinely observed (Figure 1B) with approximately 50% of *Aire*-deficient mice progressing to severe staining by 16 weeks of age (Figure 1C). Lymphocytic infiltration of the conjunctiva in *Aire*-deficient mice was compatible with that found in conjunctival biopsy specimens from human patients with SS. CD3^+^ (pan-T cell maker) and CD4^+^ T cells have been shown to occur along with drying of the ocular surface in SS patients [33-35]. Moreover, the positive correlation between lissamine green staining and squamous biomarker, SPRR1B, in *Aire*-deficient mice (Figure 1B, Figure 3A, and Figure 6A) is in concordance with a previous human study that showed a correlation between squamous metaplasia and Rose Bengal staining using impression cytology specimens from SS patients [36]. Taken together, spontaneous, multifocal, lymphocytic infiltration of the lacrimal gland and progressive damage to the ocular surface epithelium substantiates the use of *Aire*-deficient mice to study the immunopathology of autoimmune-mediated dry eye disease.

In our investigation of the afferent arm of the immune loop, we observed an accumulation of DCs in the limbus and mobilization of activated DCs into the cornea. Resident CD11c^+^ DCs infiltrated the entire ocular surface with the most significant accumulation in the limbus and cornea (Figure 5A). This finding is similar to a previous study that showed CD11c^+^ DCs throughout the corneal stroma of inflamed eyes in Balb/c mice while in the absence of inflammation, only small populations were isolated to the anterior stroma of the central cornea [26,27]. The increased presence of resident and activated DCs in the *Aire*-deficient mouse implies the existence of autoantigen in the central cornea, either the epithelium or the stroma. The initial appearance of activated DCs in the limbus at eight weeks of age followed by a sharp elevation of cell numbers in the central cornea by 16 weeks indicates centripetal movement of local/circulating DCs into the cornea with increased activation as the mice age (Figure 5C). Although centripetal migration of DCs from the limboconjunctiva to the cornea is known to play a critical role in promoting chronic immune inflammation in models of corneal graft rejection and infection [37], it remains unclear how these professional APCs contribute to the priming and homing of autoreactive T cells in autoimmune dry eye disease. Clinically, patients with an *AIRE* mutation show DC hyperactivation and impaired maturation in terms of costimulatory cytokine secretion, which underlies altered T cell responsiveness and autoimmunity [38]. The detailed mechanism by which DCs in *Aire*-deficient mice control cell-mediated autoimmunity remains to be elucidated.

To complete the proposed immune cycle that exists in patients with autoimmune dry eye disease, an effector arm leading to lymphocytic infiltration of the ocular surface is initiated following antigen presentation to T cells in the lymph node. In the early to middle stages of *Aire*-deficient ocular surface disease (between four and eight weeks), there was dense limbal and conjunctival infiltration of predominantly CD4^+^ T cells (Figures 4A,C). In the late stage disease (between 8−16 weeks), there was a remarkable increase in CD4^+^ T cell infiltration of the central cornea (Figure 4G) with a similar pattern noted for CD8^+^ cells. Interestingly, the kinetics of effector T cell infiltration of the cornea in *Aire*-deficient mice corresponded closely to that of afferent APCs (Figure 5C). Despite the distinct nature of autoimmune dry eye disease, data presented here provide an immune loop compatible to what has been proposed in the desiccating stress model of evaporative dry eye [8]. In this model, desiccating stress breaks self-tolerance and induces CD4^+^ T cell-mediated inflammation against unknown epitopes on the ocular surface and lacrimal gland [1]. Here, we observed spontaneous CD4^+^T cell-mediated inflammation of the ocular surface and lacrimal gland (Figure 4C and Figure 1A) following deletion of *Aire*. CD4^+^ T cells are known to be the major contributors to the autoimmune response in *Aire*-deficient mice [17]. Although the specific autoantigens that are targeted in the *Aire*-deficient mouse model of SS-like disease are currently unknown, uveitis in *Aire*-deficient mice is driven by a single self-antigen [20], suggesting the possibility of Ag-specific therapies to suppress activation of autoreactive cells. Based on the data provided here and previous work in the evaporative dry eye model, there are commonalities in the immunological mechanism between autoimmune and non-autoimmune dry eye diseases. Another piece of indirect evidence supporting this “common pathological pathway” is the fact that topical cyclosporin A, an immunosuppressive agent against T cells, can be effective in treating both Sjögren and non-Sjögren dry eyes [39]. Future immunopathological studies of autoimmune and non-autoimmune dry eye may help to identify common pathways that exist along the immune loop that mediates ocular surface disease.

Given the connection between autoreactive, effector T cells and squamous metaplasia in *Aire*-deficient mice, we sought to determine whether we could forecast the onset of squamous metaplasia using effector T cells or DCs as predictors. Interestingly, the presence of resident DCs in the limbus and activated DCs in the central cornea were the strongest predictors of squamous metaplasia, using SPRR1B as a biomarker. This implies that the early stages of autoimmune-mediated keratinizing ocular surface disease are highly dependent on the centripetal movement of APCs into the cornea and their activation (Figure 7). Infiltration of CD4^+^ T cells into the limbus was the next most compelling predictor of corneal SPRR1B expression (Figure 7). This further implicated these cells as significant mediators of keratinizing ocular surface disease. Together, these events represent a complete loop of the ocular surface immune response in *Aire*-deficient mice. The robust dependence of SPRR1B on both the afferent and efferent arms of this cycle further validates its significance as a biomarker to monitor both ocular surface damage and chronic engagement of an immune-mediated inflammation. The reported findings also define an appropriate time frame for interventional studies in the *Aire*-deficient mouse that will promote the development of more effective therapies for dry eye disease.
